# Age at Menopause and Its Main Predictors among Iranian Women

**Published:** 2014-11-01

**Authors:** Fatemeh Shobeiri, Mansour Nazari

**Affiliations:** 1 Mother and Child Care Research Center, Hamadan University of Medical Sciences, Hamadan, Iran; 2 Department of Entomology, School of Medicine, Hamadan University of Medical Sciences, Hamadan, Iran

**Keywords:** Iran, Menopause, Women

## Abstract

**Background:**

Since time of menopause is influenced by a variety of racial, environmental, and physiological factors, determining age at natural menopause and its main indicators seems to be necessary. The present study attempted to determine average age at menopause and its main predictors among Iranian women.

**Materials and Methods:**

This descriptive-analytic study was carried out on 400 postmenopausal women aged 43 to 65 years attending the health centers in Hamadan, Hamadan Province, Iran, during 2013. Due to potential effects of oral contraceptive pills (OCP) on age of menopause, we considered two groups of women with and without OCP use using cluster sampling method. Data were collected through individual interviews at the health centers.

**Results:**

The findings showed significant univariate relationships between age at menopause with some baseline variables including mother’s age at menopause (p<0.001), mother and spouse with high educational level (p<0.001), passive cigarette smoking (p<0.001), weekly physical activity (p<0.001), and high family income (p<001). Adversely, smoking was associated with early menopause.

**Conclusion:**

The postmenopausal women doing intense weekly physical activity, having mothers with late menopause, having higher monthly income, and experiencing later-age pregnancy are likely to reach menopause later than their contemporaries, while smokers have an early menopause.

## Introduction

Age at menopause is naturally influenced by a variety of racial, environmental, and physiological factors (1). Even, recent genome-wide association studies have been successful in revealing genetic determinants of age at natural menopause (2-4). In fact, some women reach menopause at an early age with unknown reason, which could be the result of an inherited issue or a one-time genetic mutation. Also, age at menopause may be potentially influenced by chronological age, mother’s age at menopause, menstrual cycle characteristics, and markers of ovarian reserve (5, 6). Besides, some environmental and iatrogenic factors including cigarette smoking, chemotherapy, ovarian surgery, or damage the reproductive or endocrine system have been identified to accelerate or delay menopausal age through inducing defects in hormonal pathways such as ovarian dysfunction (7, 8).

Postmenopausal women are reported more than 15% of the population in developed countries, but this rate is 5-8% in less developed regions of the world. By 2030, the world population of menopausal and postmenopausal women is assumed to increase to 1.2 billion, with 47 million new cases each year. As women are spending more than 1/3 of their life in the postmenopausal period and the number of postmenopausal women is increasing, physical and mental health in this period are of greater importance. Identifying the factors associated with late or early menopause is necessary because age at menopause has been associated with risk for the onset of several chronic diseases such as cardiovascular diseases, breast and endometrial cancers and osteoporosis (9).

The major role of ethnicity in determining age at menopause as well as its main predictors has been assessed in different societies. It is noted that Hispanic and African-American women reach menopause earlier, while Chinese and Japanese women reach menopause later than the average Caucasian women experiencing menopause at about age 51.5 (10). However, no comprehensive information is available in age at menopause and its main determinants among Iranian women. The present study attempted to determine average age at menopause and its main predictors among Iranian women.

## Materials and Methods

This descriptive-analytic study was carried out on 400 postmenopausal women aged 43 to 65 years old attending the health centers in Hamadan, Hamadan Province, West of Iran, for general counseling or treatment of menopausal symptoms, during 2013. The study was performed according to the Helsinki declaration protocol. The objectives of the study were explained to all participants and informed consent was then obtained from them. The study was approved by the Ethical Committee of Hamadan University of Medical Sciences. Inclusion criteria were as follows: natural cessation of menses ≥12 months, not being surgically menopause and not receiving hormone therapy. Due to potential effects of oral contraceptive pills (OCP) on age of menopause, we considered two groups of women with and without OCP use using cluster sampling method. The participants were interviewed to record baseline information regarding menarche age (year), marital status (married), age of first and last pregnancy (year), number of parity, quality of menstrual cycle (regularity), duration of breast feeding (year), history of cigarette smoking, level of physical activity (hour/week), as well as socioeconomic status.

Results were reported as mean ± standard deviation (SD) for the quantitative variables and as absolute frequencies and percentages for categorical variables. Categorical variables were compared using chi-square test or Fisher’s exact test when more than 20% of cells with expected count of less than 5 were observed. Continuous variables were also compared using t test. Predictors showing a statistically significant relation with menopausal age in univariate analysis were taken for multivariate logistic regression analysis to investigate their independence as predictors. Odds ratio (OR) and 95% confidence intervals (CI) were calculated. P values of 0.05 or less were considered statistically significant. All the statistical analyses were performed using the Statistical Package for the Social Sciences (SPSS; SPSS Inc., Chicago, IL, USA) version 15.0.

## Results

Findings showed that the mean age of women was 57.4 ± 5.1 years, of which 1.3% were less than 45 years, 9.0% were aged 46 to 50 years, 29% were aged 51 to 55 years, and 60.8% were aged 56 years or more. The mean menarche age was 13.6 ± 1.3 years, the mean age at marriage was 15.8 ± 3.6 years, and the mean age at first pregnancy was 18.4 ± 3.5. The mean age of natural menopause was 49.6 ± 4.02 years and its median was 50.0 years. Among them, 17/5% were ≤45 years old, 45% were 50-46 years old, and 37/5% were ≥51 years old.

The baseline characteristics of participants in the two groups are summarized in table 1. Except for history of infertility that was more frequent in OCP non-users as well as weekly physical activity that was intense in OCP users, both groups were similar in terms of other baseline variables. The estimated mean age of menopause for the entire sample was 49.67 ± 3.83 years, ranged 39 to 60 years, while for OCP users was 49.60 ± 4.33 years and for OCP non-users was 49.61 ± 3.52 years with no significant discrepancy. Regarding association between age at menopause and other characteristics, multivariate logistic regression analysis shows positive association between age at menopause with menarche age (Beta=0.125, p=0.012) and last pregnancy age (Beta=0.258, p<0.001). Also, using t test or one-way ANOVA analyses, we could show significant univariate relationships between age at menopause and some baseline variables including mother’s age at menopause (p<0.001), mother and spouse with high educational level (p<0.001), passive cigarette smoking (p<0.001), weekly physical activity (p<0.001), and high family income (p<0.01) (Table 2). Furthermore, our data showed that age at menopause is predictable by last pregnancy age using multivariable linear regression model. Adversely, smoking was associated with early menopause.

**Table 1:** Baseline characteristics in OCP user and non-user women


Characteristics	OCP user (n = 243)	OCP non-user (n = 157)	P value

**Women age (Y)**	57.24 ± 4.96	57.69 ± 5.35	0.404
**Age at menopause (Y)**	49.60 ± 4.33	49.61 ± 3.52	0.979
**Age at menarche (Y)**	13.60 ± 1.25	13.66 ± 1.30	0.616
**Age at first pregnancy (Y)**	18.23 ± 3.36	18.71 ± 3.61	0.182
**Age at last pregnancy (Y)**	33.28 ± 5.62	33.16 ± 5.66	0.845
**Age at marriage (Y)**	15.75 ± 3.36	15.95 ± 3.95	0.562
**Mothers’ age at menopause (< 45 year)**	38 (15.6)	29 (18.5)	0.459
**Parity**	3.60 ± 0.61	3.47 ± 0.70	0.611
**Irregular menstrual cycles**	59 (24.3)	39 (24.8)	0.899
**Breast feeding > 1 year**	211 (86.8)	132 (84.1)	0.228
**History of infertility**	2 (0.8)	12 (7.6)	< 0.001*
**Cesarean section delivery **	7 (2.9)	10 (6.4)	0.052
**Married status (yes)**	190 (78.2)	117 (74.5)	0.397
**Mother with high educational level (college)**	21 (8.6)	22 (14.0)	0.152
**Spouse with high educational level (college)**	48 (19.8)	35 (22.3)	0.689
**Mother job status as employed **	4 (1.6)	7 (4.5)	0.074
**Smoking **	3 (1.2)	3 (1.9)	0.587
**Passive smoking**	105 (43.2)	55 (35.0)	0.103
**Weekly physical activity > 20 , hours/week**	100 (41.2)	52 (33.1)	0.005*
**High family income (> 1.000.000 T)**	25 (10.3)	9 (5.7)	0.139
**Number of family members **	7.35 ± 1.88	7.21 ± 2.50	0.550


*; P<0.05 and OCP; Oral contraceptive pills.

**Table 2:** Multivariable linear regression model for identifying the determinants of the age at menopause


Characteristics	P value	Beta	SE

**Age at menarche(Y)**	0.190	0.148	0.113
**Age at last pregnancy(Y)**	0.018^*^	0.065	0.027
**Mothers’ age at menopause (Y) **	< 0.001^*^	3.643	0.397
**Mother with high educational level, college**	0.263	0.320	0.285
**Spouse with high educational level , college**	0.821	0.055	0.242
**Passive smoking**	< 0.001^*^	-1.817	0.313
**Weekly physical activity > 20 , hours/week**	< 0.001^*^	0.780	0.202
**High family income (> 1.000.000 T)**	0.002^*^	0.806	0.256


*; P<0.05.

## Discussion

The mean age at menopause was 49.6 ± 4.02 years according to previous studies (11). Our findings showed that the postmenopausal women doing intense weekly physical activity, having mother with late menopausal age, having higher monthly income, and experiencing later-age pregnancy are likely to reach menopause later than their contemporaries, while smokers have early menopause.

Age at menopause varies across different nations, while is shown a raise in all nations. But, this trend seems paradoxical because existence of several determinants. In this regard, different range of menopausal age and various related determinants have been reported from different countries. In a study by Dratva et al. (12), the median of menopausal age was 54 years and the major determinants of early menopause were current smoking, high body mass index (BMI), and low physical activity, while the determinant for late menopause was multiparity. In this context, the use of OC caused different effects on timing of menopause. In another study by Parazzini et al. (13), the mean age at spontaneous menopause was 51.2 years, and they also reported late menopause age was significantly associated with lower educational level, high BMI, a later age at menarche, lifelong irregular menstrual cycles, and higher parity, whereas smokers showed lower age at menopause. Nagel et al. (14) showed that increasing age at first full term pregnancy and a longer time interval until having regular menses are associated with later onset of natural menopause. Also, as compared to never smokers, current smokers were younger at menopause. High carbohydrate diet and high intake of vegetable, fiber and cereal products are inversely related to the age at natural menopause. Women with higher intake of fat, protein and meat had a delayed onset of natural menopause. Meschia et al. (15) reported the mean age at menopause is 50.9 years. Also, they showed that women smoking had a lower mean age of menopause than non-smokers. Moreover, a low body mass index (BMI) and an early age at menarche were related to early menopause in the crude analysis. In Bromberger’s observation, median age of menopause was 51.5 years for the whole sample. Median age at menopause were earlier for women who were African-American, smokers, and underweight, as well as who experienced irregular menstrual cycles (16). In our survey, the mean age at menopause was 49.67 and its median was 50.0 years that is consistent with the finding of other studies conducted in Iran that were included different sources of patients (17-21). A study has showed that 1% of women reach menopause by age 40, 10% by age 46, and 90% by age 55.0, approximately (22). Smokers reached menopause about 1.5 years earlier than nonsmokers due to the effect of nicotine on estrogen production and catabolism (23). Our study also suggested several new variables to consider in predicting age at menopause. Our results confirmed that there is association between intense weekly physical activity and age at menopause. Menopausal age is accelerated in women who exercise excessively. Also, those with higher socioeconomic level experience later menopause. Furthermore, we showed an association between a mother’s and a daughter’s age at menopause, as shown previously. In fact, when a mother experiences menopause early, her daughter will have poor ovarian reserve between ages 35 and 49 (24-26). This commonality may be due to some genetics variations. Genome-wide association studies (GWAS) have been successful in showing genetic determinants of age at menarche and age at natural menopause. More than 30 novel genetic loci have been identified in GWAS for age at menarche and 17 for age at natural menopause (27). Also, newly genetic hypothesis is supported by twin studies that attribute 63% of the concordance to shared genetic material. Pedigree analyses have uncovered a potential dominant pattern of inheritance of early menopause and premature ovarian failure through maternal or paternal relatives (28, 29). In total, identifying markers predicting age at menopause would be of significant value to women and clinicians. Time to menopause could influence treatment choice (medical or surgical) for women with disorders of menstruation. However, no single test can predict age at menopause, sum of these determinants should be considered to optimize predicting age at menopause. Limitation of this study should be discussed. The women analyzed were part of a large study whose main goal was to describe the characteristics of women attending health centers located in a city of Iran. Thus, they cannot be considered as a representative population of the Iranian women. However, the general characteristics of this population are similar with those of Iranian women.

## Conclusion

In this study, physical activity, education, mother’s age at menopause, last pregnancy age, income and smoking are found to be related with age at natural menopause. Our findings showed that the postmenopausal women doing intense weekly physical activity, having mother with late menopausal age, having higher monthly income, and experiencing later-age pregnancy are likely to reach menopause later than their contemporaries, while smokers have early menopause. This result would help in the planning of health services in menopause units as a new, important issue in developing countries like Iran. Additionally, these services should be available for all women at menopause age.
